# Delayed thoracodorsal nerve denervation for animation deformity following latissimus dorsi flap breast reconstruction: An 11-year retrospective analysis

**DOI:** 10.1016/j.jpra.2025.06.006

**Published:** 2025-06-14

**Authors:** Chloe Jordan, Krzysztof Sosnowski, Rushabh Shah, Kai Yuen Wong, Sarah Benyon, Michael Irwin, Charles Malata

**Affiliations:** a Department of Plastic and Reconstructive Surgery, Addenbrooke’s Hospital, Cambridge University Hospitals NHS Foundation Trust, Hills Rd, Cambridge United Kingdom; b School of Clinical Medicine, University of Cambridge, Hills Rd, Cambridge, United Kingdom; c Cambridge Breast Unit, Addenbrooke’s Hospital, Cambridge University Hospitals NHS Foundation Trust, Hills Rd, Cambridge, United Kingdom; d Anglia Ruskin University School of Medicine, Anglia Ruskin University, East Rd, Cambridge, United Kingdom

**Keywords:** Animation deformity, Thoracodorsal nerve denervation, Latissimus dorsi flap, Breast reconstruction

## Abstract

**Introduction:**

Symptomatic animation deformity (AD) following latissimus dorsi (LD) flap breast reconstruction, though uncommon, significantly impacts patient satisfaction. Primary denervation is typically avoided due to potential intraoperative risk of damage to the LD vascular pedicle and subsequent muscle atrophy, which may necessitate future fat grafting. This study evaluates the incidence of troublesome AD requiring surgical intervention and assesses the safety and efficacy of selective delayed denervation in symptomatic patients at a UK university hospital.

**Materials and methods:**

Patients who underwent LD flap breast reconstruction between January 2014 and December 2024 were retrospectively analysed. Those with troublesome AD who subsequently underwent delayed denervation were identified. Data on demographics, surgical details, and outcomes were collected to evaluate the effectiveness and safety of delayed thoracodorsal nerve denervation.

**Results:**

Among 90 LD flaps in 84 patients (78 unilateral, 6 bilateral, mean age = 53.6 years, BMI = 25.8), 8.3 % (*n* = 7) reported troublesome AD, with almost half (*n* = 3) also experiencing pain. Delayed denervation was performed in all seven, with symptom improvement in six: complete resolution in 57.1 % (*n* = 4), partial relief in 28.7 % (*n* = 2), whilst one patient required redo surgery. No complications (e.g., infection, muscle atrophy, implant loss, rippling or long thoracic nerve damage) were reported.

**Conclusion:**

Our findings demonstrate that selective delayed thoracodorsal nerve denervation effectively manages symptomatic AD while avoiding overtreatment in asymptomatic patients. This approach preserves muscle volume, unlike primary denervation, and achieves a high success rate with minimal morbidity. Future prospective studies are required to establish standardised assessment criteria and optimal timing for intervention.

## Introduction

The latissimus dorsi (LD) myocutaneous flap remains a cornerstone of chest and breast reconstruction, be it immediate, delayed or salvage, despite advancements in alternatives such as implants, acellular dermal matrices and free flaps. However, a significant medium-to-long term complication is animation deformity (AD), reportedly affecting 36–70 % of patients,^1^^,^^2^ where involuntary muscle contractions during arm movements cause breast distortion, chest tightness, or pronounced twitching with or without pain, leading to functional and aesthetic concerns. One effective treatment for this is thoracodorsal nerve (TDN) denervation.

The timing of denervation, however, remains contentious. While some advocate immediate division during initial surgery, our institution favours selective delayed denervation. Primary denervation risks vascular pedicle damage (intraoperatively) or subsequent irreversible muscle atrophy**.** Conversely, delayed denervation targets only symptomatic patients, thus avoiding surgery in asymptomatic individuals. This retrospective study evaluates outcomes of delayed denervation at a UK tertiary centre, analysing incidence, efficacy and complications. By comparing our results to existing literature, we aim to clarify the role of delayed denervation to help guide evidence-based management.

## Methods

All patients who underwent LD flap breast reconstruction (January 2014–December 2024) at Addenbrooke’s University Hospital were identified from EPIC (electronic patient record), and those who required delayed thoracodorsal denervation were evaluated. Data collected included demographics, surgical details (indications, techniques, nerve histology), and complications. All data were anonymised and statistically analysed to evaluate the efficacy and safety of delayed TDN denervation. This research was performed as part of Clinical Project number ID6686 PRN12686 of the Hospital’s Audit and Clinical Governance Department.

## Surgical technique

At the time of the initial breast reconstruction, the LD muscle insertion was transected, and the muscle was inset into the breast footprint using 2/0 PDS sutures to the pectoralis fascia, primarily along the superior and medial borders.

For optimal access during denervation, patients were positioned in the lateral decubitus position with the arm and elbow flexed at 90 degrees and the forearm resting in a supportive gutter. An incision was made along the lateral part of the pre-existing LD flap scar, extending into the axilla, or via a separate axillary skin crease incision. Dissection exposed the anterolateral border of the LD muscle, and the thoracodorsal nerve was identified deep to it. The nerve was then separated from the vascular pedicle and, prior to division, was confirmed using a nerve stimulator. It was transected, with a 1–2 cm segment excised, and in some cases, ligaclips were applied to prevent regeneration. Haemostasis was achieved and the wound closed in layers.

## Results

Over 11 years, 84 patients underwent 90 LD flap breast reconstructions. Patient demographics are summarised in Table 1. Postoperatively, 7 patients (8.3 %) reported animation causing significant cosmetic distortion, with three of them also experiencing pain. Six of these seven patients had undergone reconstruction in conjunction with implants placed in the prepectoral plane. None demonstrated signs of capsular contracture, such as palpable implant distortion or firmness. All patients underwent unilateral delayed TDN denervation under general anaesthetic (see Table 2). Excised nerve segments were histologically confirmed in all patients.

**Table 1:** Patient demographics and clinicopathological details of all patients undergoing LD flap breast reconstruction.

Age	53.63 (SD 11.5)
BMI	25.83 (SD 4.3)
Smoking status	
Current smokers	2 (2.4 %)
Ex-smoker	29 (34.5 %)
Never smoked	53 (63.1 %)
Comorbidities	
Ischaemic heart disease	21 (25.0 %)
Diabetes	2 (2.4 %)
Autoimmune	3 (3.6 %)
Obesity	7 (8.3 %)
Immediate vs delayed	
Immediate	70 (77.7 %)
Delayed	20 (22.2 %)
Unilateral vs bilateral	
Unilateral	78 (92.9 %)
Bilateral	6 (7.1 %)
Risk reducing vs therapeutic	
Therapeutic	80 (88.9 %)
Risk reducing	10 (11.1 %)

**Table 2:** Demographics and outcomes for patients undergoing thoracodorsal nerve denervation.

Age	Smoking history	Comorbidities	Laterality	Immediate/ delayed	Reason	Anaesthesia type	Length of excised nerve	Histology results	Symptoms Post-surgery	Revision	Complications
53	Ex-smoker	Autoimmunity	Left	Delayed	Animation	GA	1cm	Nerve tissue	Complete resolution	No	None
60	Non-smoker	Cardiovascular	Left	Delayed	Pain and animation	GA	1cm	Nerve tissue	No improvement	Yes	Yes – revision operation resulting in complete symptom resolution
60	Non-smoker	Cardiovascular	Left	Delayed	Animation	GA	1cm	Nerve tissue	Partial resolution (some intermittent twitching)	No	None
38	Non-smoker	Obesity	Right	Delayed	Animation	GA	2cm	Nerve tissue	Complete resolution	No	None
70	Non-smoker	Obesity	Left	Delayed	Animation	GA	1.5cm	Nerve tissue	Complete resolution	No	None
43	Non-smoker	N/A	Right	Delayed	Pain and animation	GA	Not excised: nerve was divided	N/A	Partial resolution (some intermittent twitching)	No	None
41	Ex-smoker	N/A	Right	Delayed	Pain and animation	GA	1cm	Nerve tissue	Complete resolution	No	None

Complete symptom resolution occurred in 4 patients (57.1 %), partial relief in 2 (28.7 %), whilst one required redo denervation for full resolution. No early or late complications such as muscle atrophy requiring fat grafting arose, yielding an 85.7 % success rate.

## Discussion

The LD flap is a well-established option for breast reconstruction, yet animation deformity (AD), an underreported complication, lacks consensus on optimal management. Reported AD incidence varies widely (36–70 % of patients), though our study demonstrated a markedly lower rate (8.33 %).^1^^,^^2^ This discrepancy may reflect differences in definitions.

While some advocate prophylactic denervation during initial flap reconstruction, long-term outcomes remain poorly characterised. Immediate denervation risks injury to the main vascular pedicle, and can cause subsequent muscle atrophy necessitating fat grafting to correct volume loss, a complication totally avoided in our delayed cohort. While delayed denervation poses technical challenges, because of fibrosis and scarring from flap harvest and axillary cancer surgery,^3^ our outcomes demonstrate its safety and efficacy: 85.7 % of denervated patients (6/7) achieved symptom resolution without complications, in keeping with studies favouring selective intervention.^4^ Notably, one patient required revision denervation, a rarely reported scenario, underscoring the importance of meticulous intraoperative nerve identification.

While prior studies recommend excising >4 cm of the thoracodorsal nerve, we achieved complete AD resolution with 1–2 cm segments, suggesting adequate denervation can be achieved conservatively. Additionally, no patients required fat grafting post-denervation, contradicting murine models linking denervation to muscle atrophy,^5^ and supporting studies in which denervation timing did not correlate with volume loss.

This study’s major limitation is the retrospective design and small denervation cohort. The incidence of AD may be underrepresented due to selection bias, as asymptomatic patients were not systematically assessed. Larger trials with standardised AD metrics and postoperative follow-up are essential to validate our findings.

## Conclusion

Delayed TDN denervation offers a safe and effective solution for troublesome AD following LD breast reconstruction, avoiding overtreatment in asymptomatic patients whilst probably preserving muscle volume. Our results challenge technical norms, demonstrating efficacy with shorter nerve resections, hence, we advocate selective intervention guided by patient symptoms. Future prospective studies should evaluate long-term outcomes and help develop standardised guidelines to manage patients with problematic AD.

## Conflicts of interest

None declared.
